# Impact of Sex on Viral Shedding and Symptom Severity During Acute COVID-19

**DOI:** 10.20411/pai.v11i1.971

**Published:** 2026-05-06

**Authors:** Evelyn Kung, Rinki Deo, Manish C. Choudhary, Kara W. Chew, Teresa H. Evering, Rachel Bender Ignacio, Prasanna Jagannathan, James P. Flynn, James Regan, Carlee Moser, Mark J. Giganti, Michael D. Hughes, Justin Ritz, Arzhang Cyrus Javan, Alexander L. Greninger, Upinder Singh, William Fischer, Eric S. Daar, David A. Wohl, Joseph J. Eron, Judith S. Currier, Robert W. Coombs, Davey M. Smith, Jonathan Z. Li, for the ACTIV-2/A5401 Study Team

**Affiliations:** 1 Brigham and Women's Hospital, Harvard Medical School, Boston, Massachusetts; 2 David Geffen School of Medicine, University of California Los Angeles, Los Angeles, California; 3 Weill Cornell Medicine, New York, New York; 4 University of Washington, Seattle, Washington; 5 Fred Hutch Cancer Center, Seattle, Washington; 6 Stanford University School of Medicine, Stanford, California; 7 Harvard T.H. Chan School of Public Health, Boston, Massachusetts; 8 NIH Division of AIDS (DAIDS), National Institutes of Health, Rockville, Maryland; 9 University of Washington, Seattle, Washington; 10 University of North Carolina at Chapel Hill School of Medicine, Chapel Hill, North Carolina; 11 Lundquist Institute at Harbor-UCLA Medical Center, Torrance, California; 12 University of California, San Diego, California

**Keywords:** Sex Differences, Acute SARS-CoV-2 Infection, Nasal Viral RNA Shedding, Days Since Symptom Onset, Symptom Dynamics

## Abstract

**Background:**

To evaluate the impact of sex on acute SARS-CoV-2 infection, 668 participants from the ACTIV-2/A5401 study were followed over a 28-day period.

**Methods:**

A primary analysis was performed on 469 participants with quantifiable viral loads at baseline.

**Results:**

Male and female participants had comparable nasal SARS-CoV-2 RNA levels at study entry and throughout follow-up. However, sex-specific differences in viral shedding emerged when stratified by symptom duration. In the first 3 days after symptom onset, female participants exhibited higher nasal SARS-CoV-2 RNA levels than males, but lower viral RNA levels thereafter. The higher viral RNA levels in females during the earliest phase of acute COVID-19 were observed even after adjusting for age, race, and region of enrollment. Female participants also tended to have higher symptom scores across days since symptom onset, but no significant correlation was observed between nasal SARS-CoV-2 RNA levels and symptom score regardless of sex.

**Conclusion:**

These findings highlight the impact of sex on both viral shedding and symptom dynamics and underscore the importance of considering time since symptom onset when evaluating antiviral therapies for respiratory viruses in clinical trials.

## INTRODUCTION

The COVID-19 pandemic, caused by Severe Acute Respiratory Syndrome Coronavirus 2 (SARS-CoV-2), has had an unprecedented impact on global mortality [1]. Previous research has established the relationship between older age and the presence of comorbidities (eg, diabetes, hyper-tension) with increased COVID-19 morbidity and severity [1–3]. Prior studies also suggest that biological sex influences immune responses and clinical manifestations in COVID-19, but its role in shaping viral shedding dynamics during acute infection remains incompletely understood [4–6]. It has been well-documented that male individuals are at greater risk of severe COVID-19. One analysis reported that intensive care unit admission rates in male patients were twice as high as in females, identifying male sex as an independent risk factor for mortality [7]. Similarly, a study of hospitalized patients in the United Kingdom showed that males were placed in more severe symptom categories compared to females [8]. How SARS-CoV-2 RNA decay and symptom severity during acute mild-to-moderate COVID-19 might differ by sex would benefit from further exploration. Prior work in the ACTIV-2/A5401 study reported intriguing findings, including potential differences in SARS-CoV-2 RNA levels at baseline by race and differences in viral decay by sex [9]. In this study, we expanded upon this analysis by including additional placebo recipients of ACTIV-2/A5401, performing a concurrent analysis of how symptom scores differ by sex, and evaluating how our findings differed when analyzed by study day (as most SARS-CoV-2 treatment trials do) and also by days since symptom onset.

## METHODS

ACTIV-2/ACTG (Advancing Clinical Therapeutics Globally) A5401 is a randomized, placebo-controlled platform trial designed to evaluate the safety and efficacy of investigational agents for the treatment of non-hospitalized adults with mild-to-moderate COVID-19 [9, 10]. The analysis included data from 668 participants enrolled between August 27, 2020, and August 31, 2021, who received a placebo in the ACTIV-2/A5401 study and were recruited from treatment centers across 6 countries (United States, Brazil, South Africa, Mexico, Argentina, and the Philippines). Participants were symptomatic non-hospitalized adults aged ≥18 years. We performed a primary analysis of 469 participants with quantifiable baseline SARS-CoV-2 viral loads and a secondary analysis that included participants who had undetectable viral loads at study entry.

All participants were enrolled in the study within 11 days since symptom onset (DSSO). The protocol was approved by a central institutional review board, Advarra (Pro00045266), and participants provided written informed consent before undergoing study procedures. All participants self-collected anterior nasal (AN) swabs on days 0, 3, 7, 14, and 28 for quantitative SARS-CoV-2 RNA testing as previously described [9, 11]. Viral decay during the early phase of infection was estimated using longitudinal viral load measurements from study entry up to, but not including, the first visit at which viral load fell to ≤ lower limit of quantification (LLOQ) (1.7 log_10_). Participants with fewer than 2 viral load measurements within this interval were excluded from slope estimation. For each participant, viral decay was quantified by fitting a linear regression of log_10_ (viral load) vs study day, with the resulting slope used as a participant-level summary measure of viral load decline (log_10_ units per day).

Total symptom scores were calculated based on a 29-day diary completed by participants for 13 targeted symptoms [10]. The targeted symptoms were feeling feverish, cough, shortness of breath or difficulty breathing, sore throat, body pain or muscle pain or aches, fatigue, headache, chills, nasal obstruction or congestion, nasal discharge, nausea, vomiting, and diarrhea. Each symptom was recorded daily by the participant as absent (score 0), mild (1), moderate (2), or severe (3). Total symptom score was calculated for a given day by summing the scores for the 13 symptoms (possible range of 0-39).

Categorical variables were summarized using frequencies and percentages, and between-group differences were evaluated using either chi-squared test or Fisher's exact test as appropriate. Continuous variables were summarized with median and interquartile ranges and compared with non-parametric methods (Wilcoxon rank-sum test to compare two groups. R (4.3.0) was used for statistical analyses. Two-tailed tests were used for all the analyses, and *P*<0.05 was considered statistically significant.

## RESULTS

A total of 469 ACTIV-2/A5401 trial participants who received placebo were studied, including 220 males (47%), 249 females (53%), 210 White (45%), 41 Black (9%), and 183 Hispanic (39%) patients. The distribution of SARS-CoV-2 variants was similar between male and female participants (male: Alpha 15%, Delta 18%, Other 68%, female: Alpha 16%, Delta 18%, Other 66%). The median (Q1, Q3) age for males was 48 years (38.75, 57), with females having a similar distribution of 48 (36, 55). Male and female participants had similar DSSO at the time of enrollment (male vs female, median [Q1, Q3]: 5.5 [3, 7] vs 6 [4, 7]). Further participant demographics can be found in Table 1.

**Table 1.** Demographic Characteristics of Participants Stratified by Sex

Characteristic	Male (n=220)	Female (n=249)	*P* value
**Median age (Q1, Q3)**	48 (38.75, 57)	48 (36, 55)	0.24
**Country, n (%)**			
ARG	13 (5.6)	19 (7.6)	
BRA	8 (3.6)	8 (3.2)	
MEX	1 (0.45)	1 (0.40)	0.93
USA	181 (82.3)	199 (79.9)	
ZAF	17 (7.7)	22 (8.8)	
**Symptom score at study entry** (Q1, Q3)	9 (6, 13)	11 (7, 16)	<0.01
**Median AN SARS-CoV-2 viral load at study entry in *log_10_ copies/mL* (Q1, Q3)**	4.96 (3.87, 6.48)	5.18 (3.74, 6.35)	0.91
**Median days from symptom onset to study entry (Q1, Q3)**	5.5 (3, 7)	6 (4, 7)	0.36

Statistical analyses were performed using Wilcoxon rank-sum tests for quantitative variables and chi-squared tests for categorical variables. Abbreviations: ARG, Argentina; BRA, Brazil; MEX, Mexico; USA, United States of America; ZAF, South Africa; AN, Anterior Nasal

**Figure 1.**
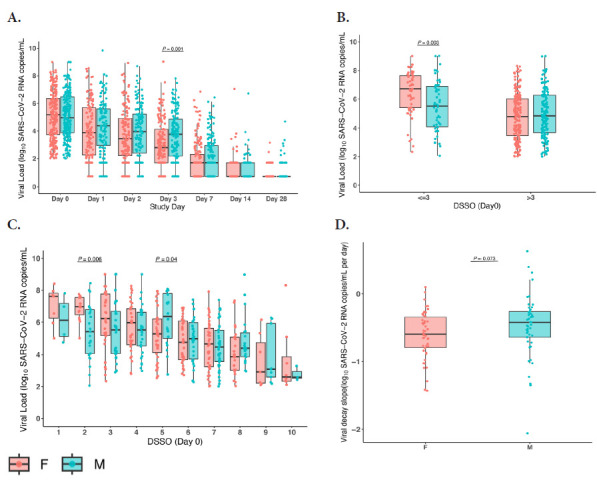
**Nasal SARS-CoV-2 viral load (VL) comparisons between males and females.** (A) Viral load by study day. (B) Viral load at enrollment (Day 0) stratified by ≤3 vs >3 days since symptom onset (DSSO). (C) Viral load at enrollment (Day 0), further categorized by individual DSSO days (1–10). (D) Viral decay at DSSO Day 0. Boxplots represent the interquartile range (IQR; 25th–75th percentile), with the horizontal line indicating the median. Individual data points are overlaid as dots. *P*-values were calculated using Wilcoxon rank-sum tests.

We first performed an analysis of SARS-CoV-2 viral load by study day for participants with detectable viral load at baseline. There were no significant differences in the proportion of male vs female participants with viral load <LLOQ at the time of study entry (29% female vs 31% male, *P*=0.43). While male and female participants exhibited similar baseline levels of nasal SARS-CoV-2 RNA levels at study entry, females had a significantly higher viral load at study day 3 (*P*<0.01; Figure 1A). There were no significant differences in viral load by sex at study days 7, 14, or 28. However, analysis by study day does not account for the timing of symptom onset for each participant. When nasal RNA levels at the time of study entry were analyzed by accounting for DSSO, we did note that early in the disease course (≤3 DSSO), there was a difference in the nasal viral RNA levels by sex: female participants had significantly higher viral loads than male participants at study entry (female vs male: median 6.7 vs 5.5 SARS-CoV-2 RNA log_10_ copies/mL, *P*<0.01; Figure 1B). Later from symptom onset (≥5 DSSO), levels of SARS-CoV-2 were generally similar or lower in female compared to male participants (Figure 1C). We therefore performed an exploratory post hoc analysis using a cutoff of ≤3 DSSO to capture the earliest symptomatic phase of infection, based on the day-by-day DSSO pattern and the expectation that viral burden is highest early in acute infection.

In a multivariable analysis of participants who entered the study ≤3 DSSO, female participants had higher viral RNA levels (*P*=0.004) after adjusting for age, race, and geographic region of enrollment (Supplementary Table 1). We also performed a secondary analysis of all participants (668), including those with undetectable SARS-CoV-2 RNA levels at study entry. Similar trends in higher viral loads in female participants earlier after study entry (≤3 DSSO) were also ob-served (Supplementary Figure 1). To further evaluate whether these differences translated into differences in viral clearance, we next assessed early viral decay rates. To assess early viral decay, we focused on the 111 participants enrolled within ≤3 days of symptom onset. Of these, 3 were excluded because they had fewer than 2 viral load measurements within the eligible interval for slope estimation, leaving 108 participants for analysis.

Viral decay showed a trend toward faster decline in female compared with male participants, although this difference did not reach statistical significance (median viral decay for females vs males: -0.59 vs -0.42 log_10_/day, *P*=0.07; Figure 1D).

**Figure 2.**
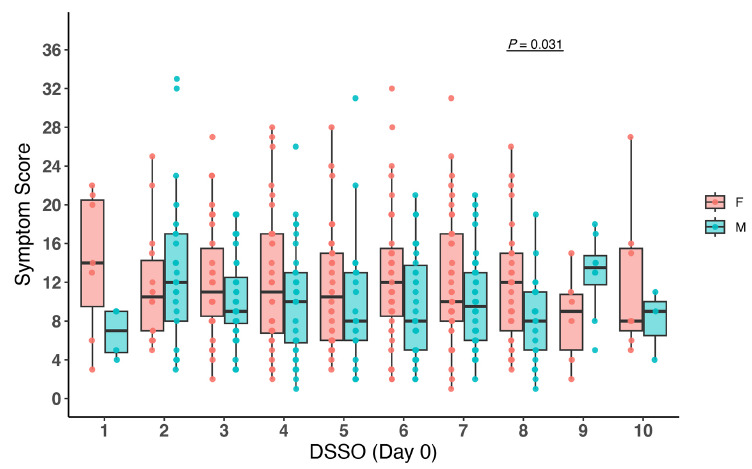
**Comparison of male and female symptom scores by days since symptom onset (DSSO).** Data are shown as boxplots representing the interquartile range (IQR; 25th to 75th percentile) with the median as a horizontal line within the box. Individual data points are shown as dots. *P*-values were calculated using Wilcoxon rank-sum tests.

**Figure 3.**
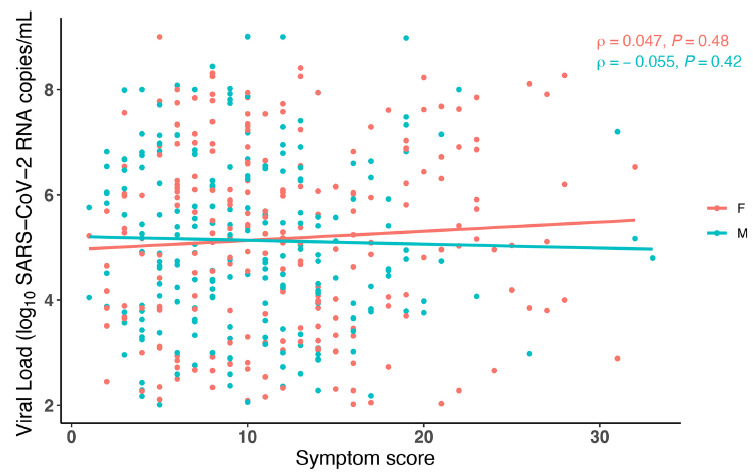
**Comparison of Symptom Score (SS) and nasal SARS-CoV-2 viral load (VL) stratified by sex.** Each dot represents an individual participant. Simple linear regression was used to generate lines of best fit, and Spearman's rank correlation was performed to assess statistical significance. *P*-values less than 0.05 were considered significant.

Similar to the viral load analysis above, we also analyzed symptom scores by sex at study entry, stratified by DSSO. In general, female participants had higher levels of symptom scores across DSSO (Figure 2). The results were similar in the secondary analysis that included participants with undetectable nasal viral RNA at study entry (Supplementary Figure 2). We also analyzed the relationship between viral load and symptom score and found no significant correlation between SARS-CoV-2 RNA levels and symptom severity by sex (Figure 3) at the time of study entry.

## DISCUSSION

This study examined the relationship between sex and levels of nasal SARS-CoV-2 RNA and symptom severity. Using data from a rigorously performed clinical trial (ACTIV-2) that included a quantitative SARS-CoV-2 viral load assay, we found that sex-specific differences in viral shedding were more evident when considering duration of symptoms than by study day alone. In the first 3 days after symptom onset, female participants had higher nasal SARS-CoV-2 viral loads compared to men. We also found that women generally had higher symptom scores at study entry regardless of DSSO, but that there were no significant correlations between nasal SARS-CoV-2 RNA levels and symptom score regardless of sex. A secondary analysis of all participants, including those with undetectable SARS-CoV-2 RNA levels at study entry, demonstrated concordant results.

In the field of virology, numerous examples illustrate how biological sex may impact viral infections. In HIV, female sex has been found to impact the extent of plasma viremia, which is likely modulated by the estrogen hormone and its effects on T cell activation and HIV reservoir activity [12, 13]. In the influenza field, human challenge experiments have shown that women appear to have a greater number of symptoms than men, although the number of days of viral shedding did not differ by sex [14]. Studies of COVID-19 have reported disparate results, with some reporting sex-based differences in viral shedding [15, 16], while others have not [17]. However, strengths of this study include the rigorous clinical trial design and the use of a quantitative SARS-CoV-2 viral load assay, which is not readily available outside of the clinical trial setting. A prior study of ACTIV-2 clinical trials participants had reported no significant difference in nasal SARS-CoV-2 RNA levels at study entry between female and male participants, although there appeared to be a greater decline in viral RNA in female participants [9]. Our results underscore the importance of accounting for the timing of symptom onset when analyzing viral shedding and symptoms, given the wide range of symptom durations among trial participants at study entry.

In addition to its role in the female reproductive cycle, estrogen is a key regulator of inflammation within the respiratory tract and modulates both the innate and adaptive immune responses [18, 19]. Intriguingly, there is also evidence that the SARS-CoV-2 spike protein binds the estrogen receptor, inducing estrogen-receptor-dependent biological effects and potentiating estrogen effects in alveolar macrophages and other cell types [20]. Prior studies have reported discordant findings on the relationship between concurrent SARS-CoV-2 RNA shedding and symptom scores [21, 22], although a human challenge experiment found no correlation between viral load and symptoms [23]. We have now extended those findings to show no significant correlation between viral RNA levels and symptom scores in both female and male participants.

Limitations of this study include that participants were enrolled largely during the early phases of the COVID-19 pandemic. In addition, because these participants were enrolled before the emer-gence of Omicron and amid evolving host immunity, it is uncertain how generalizable these find-ings are to later phases of the pandemic. It is also unclear why a subset of individuals had SARS-CoV-2 RNA levels below the limit of quantification at baseline. Participants were eligible for study enrollment if they had documented laboratory-confirmed SARS-CoV-2 infection by a molecular or antigen test from a respiratory specimen obtained prior to study entry. It is possible that some participants had rapid viral clearance before study enrollment, although we found no significant differences in the proportions of females and males with undetectable viral RNA at baseline, and a secondary analysis including all participants yielded concordant results. An additional limitation is that we assessed upper respiratory tract viral load and symptom burden during early, non-hos-pitalized infection, but did not measure lower respiratory tract viral burden or inflammatory responses, which may be more directly related to severe outcomes and may differ by sex. Future analyses could also incorporate a more detailed evaluation of post-entry time points by DSSO, including analyses of intervention-arm participants.

In summary, our analysis of a rigorously performed clinical trial highlights sex-based variations in nasal viral RNA shedding and symptom scores. These results emphasize the importance of considering time since symptom onset when analyzing clinical trials of antiviral treatments and suggest the need for further research to better understand how estrogen and sex influence the progression and outcomes of respiratory tract infections.

## SUPPLEMENTARY DATA

Supplementary materials are available at the *Pathogens and Immunity* website. Supplementary data may be provided by the authors to benefit the reader. Supplementary data are not copyedited and are the sole responsibility of the authors. Questions or comments related to supplementary materials should be addressed to the corresponding author.
