# Supplementary material for: Impact of Sex on Viral Shedding and Symptom Severity During Acute COVID-19
Source: Pathog Immun. 2026 May 6;11(1):142–53. doi: 10.20411/pai.v11i1.971 (PMC13182785; doi:10.20411/pai.v11i1.971)
Supplement: Supplementary Tables and Figures [file pai-11-142-s01.pdf]

Supplementary Table 1. Multivariate Analysis of SARS-CoV-2 Viral Load at Study Entry in Those with Quantifiable Results and ≤ 3 Days From Symptom Onset

Variable	β	95% CI	p-value
**AGE**	0.00	-0.02, 0.03	>0.9
**SEX - F**	—	—	—
**SEX - M**	-0.92	-1.5, -0.30	0.004
**Race - BLACK**	—	—	—
**Race - HISPANIC**	-0.60	-2.2, 1.0	0.5
**Race - OTHER**	1.2	-0.55, 2.9	0.2
**Race - WHITE**	-0.06	-1.6, 1.7	>0.9
**Country/Region - North America**	—	—	—
**Country/Region - South Africa**	-0.57	-2.3, 1.2	0.5
**Country/Region - South America**	0.35	-0.96, 1.7	0.6

Abbreviations: CI = Confidence Interval, β coefficient with 95% Confidence Interval

Kung E, Deo R, Choudhary MC, Chew KW, Evering TH, Ignacio RB, Jagannathan P, Flynn JP, Regan J, Moser C, Giganti MJ, Hughes MD, Ritz J, Javan AC, Greninger AL, Singh U, Fischer W, Daar ES, Wohl DA, Eron JJ, Currier JS, Coombs RW, Smith DM, Li JZ. Impact of Sex on Viral Shedding and Symptom Severity During Acute COVID-19. *Pathogens and Immunity*. 2026;11(1):142-153. doi: 10.20411/pai.v11i1.971

PATHOGENS AND IMMUNITY
IN ASSOCIATION WITH CASE WESTERN RESERVE UNIVERSITY AND THE RICHARD J. FASENMYER FOUNDATION

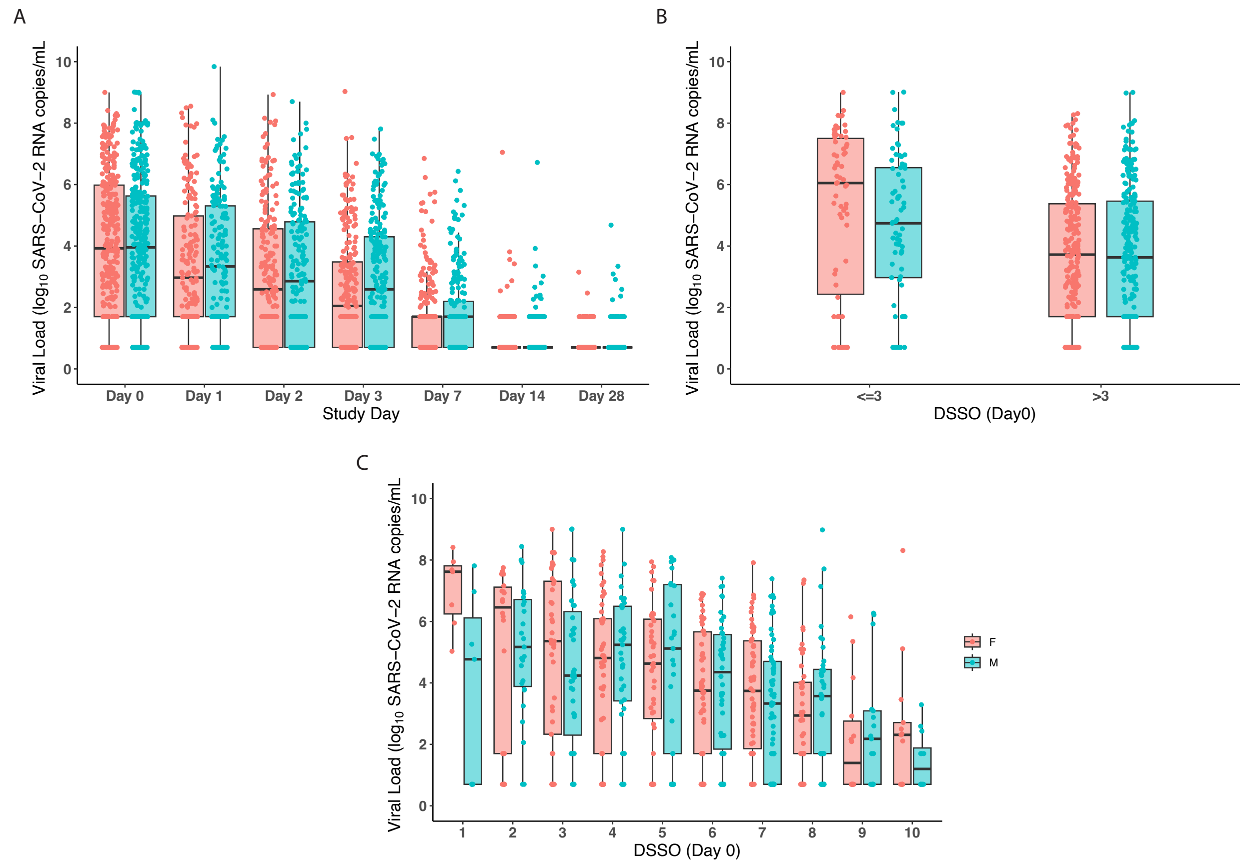

Supplementary Figure 1. Nasal SARS-CoV-2 viral load (VL) comparisons between males and females for 668 participants, including those with undetectable viral RNA levels at time of study entry. (A) Viral load by study day. (B) Viral load at enrollment (Day 0) stratified by ≤3 vs >3 days since symptom onset (DSSO). (C) Viral load at enrollment (Day 0), further categorized by individual DSSO days (1–10). Boxplots represent the interquartile range (IQR; 25th–75th percentiles), with the horizontal line indicating the median. Individual data points are overlaid as dots. *P*-values were calculated using Wilcoxon rank-sum tests.

Kung E, Deo R, Choudhary MC, Chew KW, Evering TH, Ignacio RB, Jagannathan P, Flynn JP, Regan J, Moser C, Giganti MJ, Hughes MD, Ritz J, Javan AC, Greninger AL, Singh U, Fischer W, Daar ES, Wohl DA, Eron JJ, Currier JS, Coombs RW, Smith DM, Li JZ. Impact of Sex on Viral Shedding and Symptom Severity During Acute COVID-19. *Pathogens and Immunity*. 2026;11(1):142-153. doi: 10.20411/pai.v11i1.971

PATHOGENS AND IMMUNITY
IN ASSOCIATION WITH CASE WESTERN RESERVE UNIVERSITY AND THE RICHARD J. FASENMYER FOUNDATION

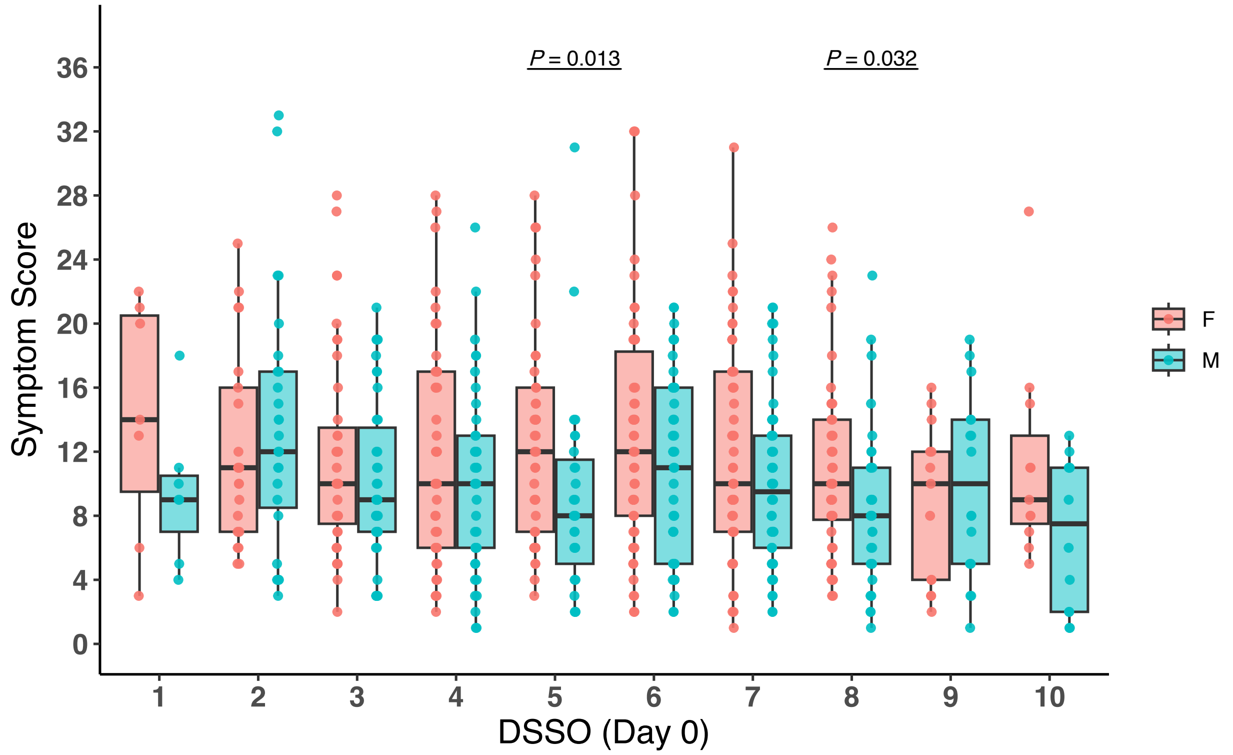

Supplemental Figure 2. Comparison of male and female symptom scores by days since symptom onset (DSSO) from secondary analysis, including all participants. Data are shown as boxplots representing the interquartile range (IQR; 25th–75th percentiles) with the median as a horizontal line within the box. Individual data points are shown as dots. *P*-values were calculated using Wilcoxon rank-sum tests.

Kung E, Deo R, Choudhary MC, Chew KW, Evering TH, Ignacio RB, Jagannathan P, Flynn JP, Regan J, Moser C, Giganti MJ, Hughes MD, Ritz J, Javan AC, Greninger AL, Singh U, Fischer W, Daar ES, Wohl DA, Eron JJ, Currier JS, Coombs RW, Smith DM, Li JZ. Impact of Sex on Viral Shedding and Symptom Severity During Acute COVID-19. *Pathogens and Immunity*. 2026;11(1):142-153. doi: 10.20411/pai.v11i1.971
